# A Simple and Practical Method for Setting Up a Criterion of Projection of Silicone Breast Implant After Simple Mastectomy

**Published:** 2017-08-18

**Authors:** Naohiro Ishii, Jiro Ando, Michiko Harao, Masaru Takemae, Kazuo Kishi

**Affiliations:** ^a^Department of Plastic and Reconstructive Surgery, Keio University, Shinjyuku Ward, Tokyo, Japan; ^b^Department of Breast Surgery, Tochigi Cancer Center, Utsunomiya City, Tochigi, Japan

**Keywords:** breast reconstruction, breast implant, projection, choice of implant, simple mastectomy

## Abstract

**Objective:** In breast reconstruction, decision of projection of silicone breast implant in tissue expander replacement is difficult because of the need to consider several parameters that cannot be expressed in accurate numerical form. The present study aimed at a quantitative analysis based on decreased projection of the reconstructed side compared with silicone breast implant projection and to develop a new method for simple and practical decision of silicone breast implant projection. **Methods:** Thirty-five patients who had mammary carcinoma and were treated with simple mastectomy, tissue expander insertion, and replacement with anatomical silicone breast implant from April 2013 to March 2016 were retrospectively identified. We recorded the projection of used silicone breast implant (Pi). The projections of reconstructed breast 6 months after silicone breast implant insertion (Pr) and that of the unaffected breast during silicone breast implant selection (Pu) were measured. The difference between Pi and Pr was defined as the revised numerical value (Rev). We investigated whether Rev significantly differed according to age, body mass index, or Pu and analyzed correlations between Rev and age, Pu, and body mass index. **Results:** Mean Rev in all patients was 1.2 ± 0.3 cm. Rev was significantly higher in patients with higher body mass index than in those with lower body mass index (*P* < .01) and in patients with higher Pu than in those with lower Pu (*P* < .01). Significant positive correlations of Rev with body mass index and Pu were found (β = .63, *P* < .01 and β = .67, *P* < .01, respectively). **Conclusions:** Rev was a simple, practical, and cost-effective concept. We believe that it is a useful indicator for deciding silicone breast implant projection.

Optimal choice of silicone breast implant (SBI) for unilateral breast reconstruction is often difficult because of the need for assessment of linear parameters and volume of the complicated shape of breast. In clinical practice, SBI is mostly chosen with reference to linear parameters of the unaffected breast or volume of injected saline in the tissue expander (TE). Recently, many researches discussed breast volume measurement using 3-dimensional (3D) imaging.^1^^-^5 However, using this technique, determining SBI projection is particularly difficult because the projection of the unaffected breast excludes the skin thickness on the affected side with TE including saline, and hence is not equal to the appropriate projection of SBI, even if the projection of the unaffected breast was equal to the projection of the affected side with TE including saline; and the difference between TE with full injected saline and SBI is found in the texture and pressure to the skin envelope. Moreover, although measuring breast volume using 3D imaging is more accurate, it is not easily performed in a general hospital because of its cost and the need for a complicated setting and operation.

If custom-made SBI is chosen by the patient, complicated measurements are needed to choose the appropriate SBI; however, at present, ready-made SBI is based only on 3 linear parameters (width, height, and projection) and volume. The factors determining SBI projection in TE replacement are projection of the unaffected breast, thickness and elasticity of the skin envelope of the affected breast with TE including saline, difference in elasticity between the unaffected breast and SBI, and thickness of the skin and subcutaneous tissue caudal to the inframammary fold (IMF) on the affected side. These factors may be related to patient's age, body mass index (BMI), and projection on the unaffected breast.

The purpose of our retrospective study was the quantitative analysis based on numerical values, namely, data concerned with thickness and elasticity of skin envelope on the affected breast with TE including saline, difference in elasticity between the unaffected breast and SBI, and thickness of the skin and subcutaneous tissue caudal to IMF on the affected side, where the projection of SBI takes the projection of the reconstructed breast. Furthermore, we investigated whether there was a significant difference between younger and older patients, patients with lower and higher BMI, and the lower and higher projection of the unaffected breast, with regard to the findings in our study, and aimed at developing a new method for simple and practical decision of projection of SBI.

## MATERIALS AND METHODS

A retrospective longitudinal evaluation of the projection of breast or SBI using linear measurement by a ruler was performed at our institution. A total of 35 consecutive patients who had mammary carcinoma, were treated by simple mastectomy, TE insertion, and replacement with anatomical and textured SBI (True form 3; Allergan, Ireland, Dublin) from April 2013 to March 2016, and had the similar projection in both the reconstructed breast and the unaffected breast were potentially eligible for the study. Patients who received postoperative radiation therapy, those with weight increase or decrease by more than 3 kg after SBI insertion, those who had definite capsule contracture (Baker's classification,^6^ ≥3) in measurement of the reconstructed breast projection, or those in whom the subcutaneous tissue caudal to the breast was too thick to measure breast projection were excluded.

**Figure 1:**
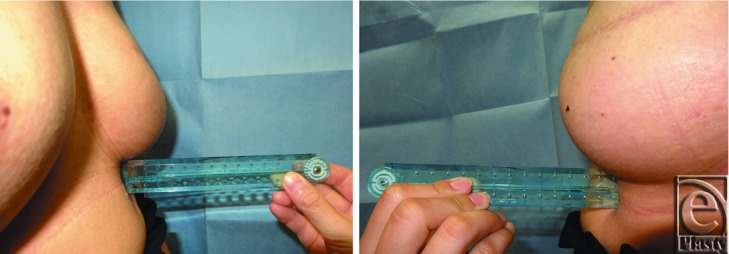
The projection of reconstructed breast was determined by measuring the distance between the chest wall and the peak of the breast with a ruler twice in medial (left) and lateral views (right) and calculating the mean for both values; same as the projection of the unaffected breast.

We recorded the projection of used SBI (Pi) and measured the projection of reconstructed breast (Pr) 6 months after SBI insertion and the projection of the unaffected breast (Pu) during SBI selection. Both Pr and Pu were determined by measuring, in lateral and medial views, the distance between the chest wall and the peak of the breast twice with a ruler and calculating the mean value (Fig 1). We compared the distribution of Pr with that of Pu in all patients.

We performed overexpansion of TE (mean ratio of expansion: 1.4) and replaced it with SBI whose projection was 0.6 to 2.0 cm higher than the projection of the unaffected breast 5 months after TE insertion. Among these cases, only those who had similarity in both Pr 6 months after SBI insertion and Pu (Pr − Pu = 0 mm or ±1 mm) were included in the study.

The first author performed all the aforementioned procedures, including TE insertion by musculofascial pocket method,^7^^,^^8^ SBI insertion under pectoralis major muscle, and measurement of breast projection.

### Revised numerical value

We defined a revised numerical value, Rev (Pi − Pr). Rev was concerned with thickness and elasticity of the skin envelope on the affected side with TE including saline, difference in elasticity between the unaffected breast and SBI, and thickness of the skin and subcutaneous tissue caudal to IMF on the affected side. For appropriate determination of Pi, Pr was the same as Pu. Consequently, optimal Pi = Pu + Rev. Furthermore, we investigated whether there was a significant difference in Rev between younger and older patients, patients with lower and higher BMI, and those with lower and higher Pu. Younger patients were younger than 50 years, whereas older patients were 50 years or older. Lower BMI was less than 21.0 (21.0: ideal BMI for Japanese), and higher BMI was 21.0 or more. Lower Pu was less than 3.5 cm and higher Pu was 3.5 cm or more. In addition, we investigated for the presence of correlations between age and Rev, BMI and Rev, and Pu and Rev, using scatter diagram in which age, BMI, or Pu were plotted on the horizontal axis and Rev was plotted on the vertical axis.

### Statistics

Data were analyzed using the Statistical Package for the Social Sciences for Windows, version 23 (IBM Corp, Chicago, Ill). Student's *t* test was used to compare continuous variables between Pr and Pu, younger and older patients, lower and higher BMI, and lower and higher Pu. Simple linear regression was used to define the linear relationship between Rev and other parameters. For all statistical tests, a *P* value of less than .05 was considered statistically significant.

The protocol for this study was approved by the institutional review board. All patients provided written informed consent before publication of this article.

## RESULTS

The characteristics of all patients included in this study are shown in Table 1. Pi, Pr, Pu, and Rev for all patients are shown in Table 2.

**Figure 2:**
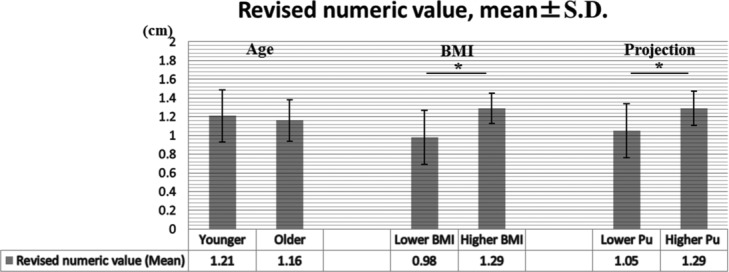
Bar chart showing comparison between Rev in younger and older patients, Rev in patients with lower and higher BMI, and Rev in lower and higher Pu. Error bars represent standard deviation. *P < .01. Rev indicates revised numerical value; BMI, body mass index; and Pu, projection of the unaffected breast.

No significant difference was found between Pu and Pr (*P* = .83). No significant difference was found in Rev between younger and older patients (*P* = .58). On the contrary, Rev in patients with higher BMI was significantly higher than in those with lower BMI (*P* < .01) and Rev in patients with higher Pu was significantly higher than in those with lower Pu (*P* < .01) (Fig 2).

**Figure 3:**
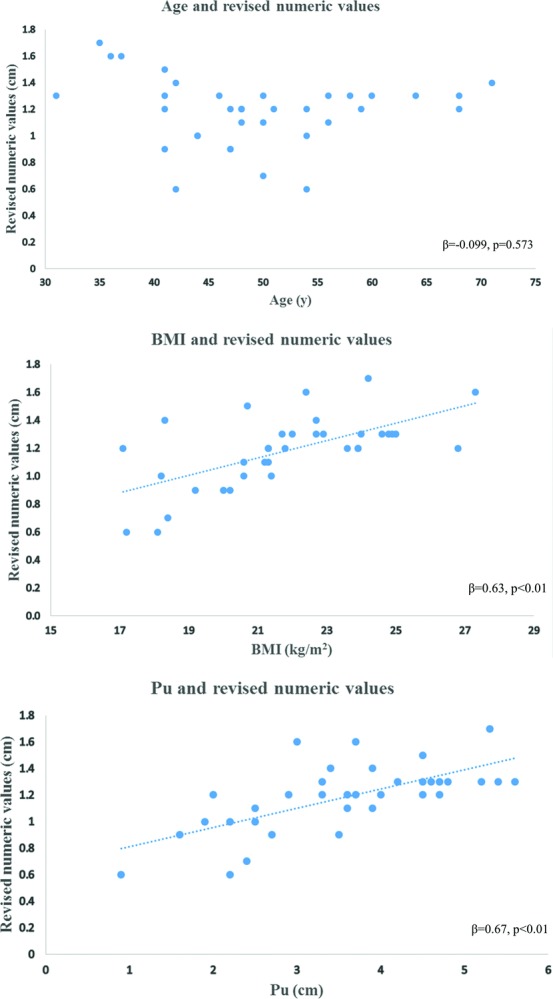
Scatter plot and simple linear regression of age and revised numerical value (a), BMI and revised numerical value (b), and Pu and revised numerical value (c). BMI indicates body mass index; Pu, projection of the unaffected breast.

Simple linear regression analysis showed no significant correlation between age and Rev (standardized regression coefficient [β] = −.0987, *P* = .573) (Fig 3*a*), whereas significant positive correlations were found between BMI and Rev (β = .63, *P* < .01) (Fig 3*b*) and between Pu and Rev (β = .67, *P* < .01) (Fig 3*c*).

Considering the significant difference in Rev between patients with lower and higher BMI and those with lower and higher Pu, and the significant positive correlations between BMI and Rev and between Pu and Rev, we showed a clinical criterion of optimal Pi as described as follows:

   Optimal Pi = Pu + 1.0 (lower BMI, < 21), Pu + 1.3 (higher BMI, ≥ 21)

   Optimal Pi = Pu + 1.1 (lower Pu, < 3.5), Pu + 1.3 (higher Pu, ≥ 3.5)

where Pi is measured in cm, Pu is measured in cm, and BMI is measured in kg/m^2^.

## DISCUSSION

This retrospective study aimed at the quantitative analysis of Rev (Pi − Pr), investigating whether there was a significant difference in Rev between younger and older patients, patients with lower and higher BMI, and those with lower and higher Pu and to test for significant correlations between Rev and age, BMI, and Pu. The results showed that no significant difference in Rev was found between younger and older patients, whereas Rev in patients with a higher BMI was significantly higher than in those with a lower BMI. Rev in patients with higher Pu was significantly higher than in those with lower Pu, and significant positive correlations were found between BMI and Rev and between Pu and Rev. These findings may be plausible because the difference in skin elasticity with aging had no significant influence on Rev. Patients with higher BMI had more thickness of the subcutaneous tissue caudal to IMF on the affected side, and patients who had higher Pu needed higher Pi; therefore, the elasticity of their skin envelope on the affected side was stronger.

Previous reports about the measurement of breast volume have shown that mammography,^9^ thermoplastic molding,^1^^,^^5^^,^^9^ magnetic resonance imaging,^1^^,^^9^ Archimedes’ principle,^9^^,^^10^ anatomical measurement,^1^^,^^5^^,^^9^^,^^11^^,^^12^ and 3D surface imaging^1^^-^5 were mainly used; and referring to reports comparing these methods, 3D surface imaging was the most accurate for the measurement of breast volume.^1^^,^^5^ Measurement of breast volume using 3D surface imaging was reported in many articles, and it has been sometimes used for preoperative SBI selection.^2^^,^^5^ However, symmetry between the reconstructed breast and the unaffected breast is not always attained when using 3D surface imaging alone to measure the linear parameters and volume of the unaffected breast, since the thickness and elasticity of the skin envelope on the affected side with TE including saline, the difference in elasticity between the unaffected breast and SBI, and the thickness of the skin and subcutaneous tissue caudal to the IMF on the affected side should be considered. Furthermore, it is not easily performed in a general hospital because of its expense and the need for a complicated setting and operation.

Morphological changes in breast projection after breast augmentation surgery were found, with the measured average values of breast projection being 21% to 25% less than expected according to the implant parameters documented by the manufacturer.^3^^,^^4^ Average decrease in projection as compared with the expected was 1.2 or 1.4 cm in round implants and 1.6 cm in anatomical implants.^3^^,^^4^ The decreased breast projection was considered to be caused by tissue attenuation of the overlying pocket or posterior displacement of the chest wall after implant insertion.^3^ However, these values were calculated by the morphological average changes alone; therefore, their distribution was not described. Furthermore, solid data about the difference between Pi and the Pr after SBI insertion following mastectomy and expander insertion had not been reported.

**Figure 4:**
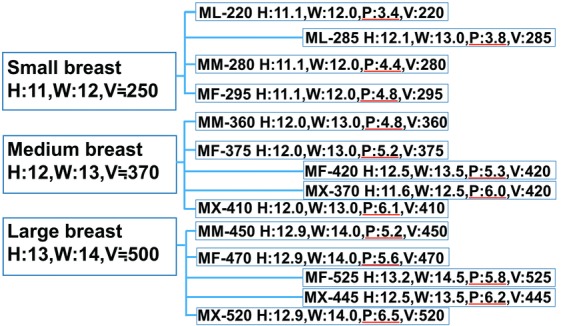
An example of the selection of SBIs in case of small, medium, and large breasts as shown by the Allergan catalogue; note that for each breast size, SBI with 3 different projections and same height and width, and with 1 to 2 intervening projections and different height and width, could be found. If a permissible range of appropriate projection of SBI was demonstrated, the decision of selection would have been easier. SBI indicates silicone breast implant; H, height (in cm); W, width (in cm); P, projection (in cm); and V, volume (in cm^3^).

SBI is ready-made and selected with consideration of 3 linear parameters (width, height, and projection) and volume. After making a guess about the volume of SBI, the width and height of SBI are first decided because of their easiness. Pi is then decided from 3 various sizes of projection with the same width and height or from the other implants (Fig 4). Projection is an important factor for the contour of the reconstructed breast; however, calculating it is often difficult. The concept of Rev is simple, practical, and cost-effective, and Rev could be easily calculated with reference to BMI and Pu. Therefore, we believe that Rev is a useful indicator for the decision of projection of SBI. Moreover, if both Rev and the breast volume using 3D surface imaging can be performed, they will be more useful indicators.

Future studies considering the various types of SBI (anatomical or round, textured or smooth, soft or hard) should be performed. In future prospective studies, Rev may be investigated in mastectomy using other methods as skin-sparing mastectomy and nipple-sparing mastectomy.

## CONCLUSION

We investigated Rev to evaluate a numerical value reflecting the thickness and elasticity of the skin envelope of the affected breast with TE including saline, the difference in elasticity between the unaffected breast and SBI, and thickness of the skin and subcutaneous tissue caudal to the IMF on the affected side. Rev in patients with higher BMI was significantly higher than in those with lower BMI and Rev in patients with higher Pu was significantly higher than in those with lower Pu and significant positive correlations were found between BMI and Rev and between Pu and Rev.

The concept of Rev is simple, practical, and cost-effective, and Rev could be easily calculated with reference to BMI and Pu. Therefore, we believe that it is a useful indicator for the decision of projection of SBI and recommend that Pi should be determined with reference to Pu and Rev based on BMI and Pu.

## Figures and Tables

**Table 1:** Patients’ characteristics*

Characteristic	
Age, mean ± SD (range), y	49.4 ± 9.7 (31–71)
Younger (<50 y), n	19
Older (≥50 y), n	16
BMI, mean ± SD (range), kg/m^2^	21.8 ± 2.6 (17.1–27.3)
Lower (<21 kg/m^2^), n	12
Higher (≥21 kg/m^2^), n	23
Pu	
Lower (<3.5 cm), n	15
Higher (≥3.5 cm), n	20
Follow-up period, mean ± SD (range), mo	21.8 ± 7.2 (12–42)

*BMI indicates body mass index; Pu, the projection of the breast on the unaffected side.

**Table 2:** The measured projection and revised numerical values in all patients

Parameters	Data	Range
Pi, mean ± SD, cm	5.1 ± 1.3	2.7-7.0
Pr, mean ± SD, cm	3.6 ± 1.1	1.7-5.4
Pu, mean ± SD, cm	3.6 ± 1.2	1.7-5.6
Rev, mean ± SD, cm	1.2 ± 0.3	0.6-1.7
Rev, mean ± 95% CI, cm	1.2 ± 0.1	

Pi indicates the projection of used silicon breast implant; Pr, the projection of the reconstructed breast; Pu, the projection of the unaffected breast; Rev, revised numerical value (Pi − Pr); and CI, confidence interval.
